# Baseline characterization of dementia cases from a reference center in Recife, Northeast Brazil

**DOI:** 10.1590/1980-5764-DN-2025-0385

**Published:** 2025-10-27

**Authors:** Mariana Albuquerque de Luna, Ana Rosa Santana, Maria Eduarda Pires, Andreia Braga Mota Azzoni, Alberto Henrique Torres Trindade da Silva, Cesar Francisco Penninck de Aguiar Kato, Bárbara Matos Almeida Queiroz, Ana Paula Silva de Oliveira, Rodrigo Cavalcanti Machado da Silva, Tatiana Caldas Neves da Silva, Simone Cristina Soares Brandão, Luziany Carvalho Araújo, Breno José Alencar Pires Barbosa

**Affiliations:** 1 Universidade Federal de Pernambuco, Hospital das Clínicas, Empresa Brasileira de Serviços Hospitalares, Serviço de Geriatria, Recife PE, Brazil.; 2 Universidade Federal de Pernambuco, Centro de Ciências da Saúde, Programa de Pós-graduação em Gerontologia, Recife PE, Brazil.; 3 Universidade Federal de Pernambuco, Hospital das Clínicas, Empresa Brasileira de Serviços Hospitalares, Serviço de Neurologia, Recife PE, Brazil.; 4 Universidade de Pernambuco, Faculdade de Ciências Médicas, Recife PE, Brazil.; 5 Universidade Federal de Pernambuco, Centro de Ciências Médicas, Recife PE, Brazil.; 6 Universidade Federal de Pernambuco, Departamento de Engenharia Biomédica, Recife PE, Brazil.; 7 Universidade Federal de Pernambuco, Hospital das Clínicas, Empresa Brasileira de Serviços Hospitalares, Serviço de Psiquiatria, Recife PE, Brazil.; 8 Universidade Federal de Pernambuco, Hospital das Clínicas, Empresa Brasileira de Serviços Hospitalares, Serviço de Terapia Ocupacional, Recife PE, Brazil.; 9 Universidade Federal de Pernambuco, Hospital das Clínicas, Empresa Brasileira de Serviços Hospitalares, Serviço de Medicina Nuclear, Recife PE, Brazil.; 10 Universidade Federal de Pernambuco, Hospital das Clínicas, Empresa Brasileira de Serviços Hospitalares, Serviço de Radiologia e Diagnóstico por Imagem, Recife PE, Brazil.

**Keywords:** Dementia, Epidemiology, Alzheimer Disease, Brazil, Demência, Epidemiologia, Doença de Alzheimer, Brasil

## Abstract

There are a limited number of reports on dementia in Brazil. Due to data heterogeneity, dementia figures in Brazil do not uniformly reflect all regions. Therefore, data from the North and Northeast regions, historically most affected by social inequalities, are required.

**Objective:**

To describe the sample of patients followed at a dementia outpatient clinic in a tertiary center in Northeastern Brazil.

**Methods:**

This was a single-center, descriptive analysis of patients evaluated in the dementia outpatient clinic at the Federal University of Pernambuco between 2018 and 2023.

**Results:**

A total of 145 patients with complete data were included in the descriptive analysis. The mean age was 71 years, and 57% were women. The most prevalent comorbidity was hypertension (54%), followed by diabetes (31%). Most patients were assessed in either the moderate (30%) or severe (33%) stage of dementia. Alzheimer disease was the most frequent diagnosis (31%), followed by frontotemporal dementia (14%) and vascular cognitive impairment (9%). Approximately 36% of the sample was classified as having mixed-pathology dementia. Neuroimaging assessments varied; 60% of patients underwent cranial magnetic resonance imaging, and molecular neuroimaging exams were performed in 42% of cases.

**Conclusion:**

Patients referred for evaluation were mostly in moderate to advanced stages of dementia, with a significant prevalence of mixed-type and non-Alzheimer’s pathologies. The present study may set the foundation for further collaborations and prospective studies.

## RESUMO.

Há um número limitado de estudos sobre demência no Brasil. Devido à heterogeneidade dos dados, os números nacionais não refletem de forma uniforme todas as regiões do país. Em especial, há escassez de dados provenientes das regiões Norte e Nordeste, historicamente mais afetadas por desigualdades sociais.

**Objetivo:**

Descrever a amostra de pacientes acompanhados em um ambulatório de demência em um centro terciário no Nordeste do Brasil.

**Métodos:**

Trata-se de uma análise descritiva, de centro único, envolvendo pacientes avaliados no ambulatório de demência da Universidade Federal de Pernambuco entre 2018 e 2023.

**Resultados:**

Foram incluídos 145 pacientes com dados completos na análise descritiva. A média de idade foi de 71 anos, sendo 57% mulheres. A comorbidade mais frequente foi hipertensão arterial sistêmica (54%), seguida por diabetes mellitus (31%). A maioria dos pacientes foi avaliada em estágios moderado (30%) ou grave (33%) da demência. A doença de Alzheimer foi o diagnóstico mais comum (31%), seguida por demência frontotemporal (14%) e comprometimento cognitivo vascular (9%). Aproximadamente 36% da amostra foi classificada como portadora de demência de etiologia mista. Os exames de neuroimagem variaram: 60% dos pacientes realizaram ressonância magnética craniana, e exames de neuroimagem molecular foram realizados em 42% dos casos.

**Conclusão:**

Os pacientes encaminhados para avaliação estavam, em sua maioria, em estágios moderados a avançados da demência, com prevalência significativa de demências mistas e não-Alzheimer. Esses achados ressaltam a importância do diagnóstico precoce e podem servir como base para futuras colaborações e pesquisas prospectivas.

## INTRODUCTION

 Approximately 50 million people currently have dementia. The rising global population age and lifestyle changes contribute to increased cases, particularly in low- and middle-income countries (LMIC)^1^. It is estimated that around 1.7 million people in Brazil are diagnosed with Alzheimer disease (AD) and other forms of dementia^2-4^. The Global Burden of Disease study revealed that the number of people with dementia worldwide more than doubled between 1990 and 2016, increasing by 117%. Among the 195 countries studied, Brazil had the second-highest age-standardized prevalence of dementia^5^. 

 The estimated global prevalence of dementia in 2050 is 150 million people^6^. While North America and Europe are expected to see a reduction in dementia incidence, lower fertility rates and increased life expectancy have led to a significant rise in cognitive disability in many LMICs, particularly in Asia, Central and North Africa, and Central and South America^7^. Control of modifiable risk factors for dementia, medical care, and education reduces the incidence of cognitive impairment in more recently born populations. However, demographic transitions marked by an aging population, rising obesity and diabetes rates, uncontrolled chronic conditions such as hypertension, and socioeconomic disparities contribute to increasing dementia cases in Latin America, Africa, and Asia^7^. 

 In Brazil, older adults (over 60) were more likely to report diabetes, hypertension, and overweight/obesity in 2015 compared to 2000-2010. In a study of the Latin American population, the global prevalence of dementia between 1980 and 2009 in individuals over 60 was higher (8.5%) than in other regions, including Europe (6.9%), North America (6.5%), and Asia (6.9%). Notably, crude prevalence varied significantly, ranging from 0.99 to 49.57%^8^. Dementia prevalence is known to increase with age, nearly doubling every five years between ages 50 and 80. It is a significant cause of disability, with a prevalence of 6.3% among individuals over 70^5^. 

 Between 1990 and 2016, deaths due to dementia increased by 148%. Dementia has become the fifth leading cause of death, following ischemic heart disease, obstructive lung disease, intracerebral hemorrhage, and ischemic stroke. It accounts for 8.6% of deaths among individuals over 70, making it the second leading cause of death in this age group^5^. In Latin America, older adults aged 65 to 69 had a higher prevalence of dementia, which may be linked to population aging, higher rates of illiteracy in this group, and inadequate management of cardiovascular risk factors. The Pietà study reported a prevalence of 21.37% among participants with no formal education, compared to 9.88% among those with at least one year of formal schooling^8^. 

 Representative samples from the North and Northeast regions, historically most affected by social inequalities, must be included. Due to data heterogeneity, dementia figures in Brazil do not uniformly reflect all regions. The Pietà study included 639 participants, revealing a high prevalence of illiteracy (30%) and low socioeconomic status among 82% of participants^8^. Another significant epidemiological study on dementia was conducted in Tremembé, reporting a 17.5% prevalence among 630 older adults in the interior of São Paulo^3,9^. In a survey carried out in Porto Alegre, the prevalence of mild cognitive impairment was 6.1%^10^. 

 The Northeast region of Brazil has higher social disparities and low literacy rates, alongside a high prevalence of clinical comorbidities that elevate the risk of dementia. Many residents face low-income conditions with limited access to public healthcare and education. The region also reports high prevalence of diabetes, hypertension, obesity, and smoking^4^. Recife is among the largest cities in the Northeast, with a population of approximately 1.6 million individuals^11^. The present study aimed to report dementia figures from a reference center in Recife and to set the foundation for further collaborations and prospective studies. 

## METHODS

 In this study, medical records of patients evaluated at the Cognitive and Behavioral Neurology outpatient clinic at Hospital das Clínicas, Universidade Federal de Pernambuco, were analyzed. All patients evaluated for suspected or diagnosed dementia from 2018 to 2023 were included. Participants routinely underwent a detailed history as well as physical and neurological examinations. Diagnoses were made according to the National Institute on Aging — Alzheimer’s Association (NIA-AA) and the Brazilian Academy of Neurology criteria^12-16^. Patients lacking essential information for clinical and epidemiological characterization, such as those with incomplete assessments or documentation errors, were excluded. Data were extracted from medical records into a Google Forms spreadsheet, including demographic information (age, education, profession, origin, marital status), comorbidities, cognitive assessment results, imaging exams, diagnosis, and follow-up time. 

### Ethical considerations

This study adhered to the National Health Council standards for research involving human subjects and was approved by the local Research Ethics Committees under protocol number CAAE 24235419.2.0000.8807. The Ethics Committees waived the requirement for written informed consent due to the use of medical record data.

## RESULTS

 One hundred sixty-two medical records were evaluated, but only 145 patients had complete data and were included in the descriptive analysis. The mean age was 71 years, with 57% women. Among the 104 caregivers, formal caregivers accounted for 11% of the total, mostly female (9 out of 11). Sons or daughters represented the largest group (54%), with a strong predominance of females (44 out of 56). Spouses comprised 35% of caregivers, predominantly male (31 out of 37). Overall, female caregivers were more common (59 out of 104, or 57%) than male caregivers (45 out of 104, or 43%). Demographic data are shown in Table 1. 

**Table 1:** Demographic data.

		Patients	Ratio (%)
**Gender**	Female	82	57
	Male	63	43
	**Total**	145	100
**Occupation** (NA=25)	Manual labor in rural areas (agriculture, mining, hunting, fishing)	10	8
	Unskilled manual labor (housework, cleaning, construction)	50	42
	Skilled manual labor (industrial production, maintenance, repairs)	9	8
	Services (retail, reception, secretarial, technical)	24	20
	Intellectual (liberal professionals, researchers, administrators)	27	23
	**Total**	120	100
**Marital status** (NA=36)	Single	5	5
	Married	11	10
	Divorced	56	51
	Widower	37	34
	**Total**	109	100
**Education level** (NA=17)	Illiterate	19	15
	1 full year	3	2
	2 to 3 years complete	9	7
	4 to 7 years complete	17	13
	8 to 11 years complete	44	34
	12 to 15 years complete	23	18
	16+	13	10
	**Total**	128	100
**Ethnicity** (NA=11)	Yellow/ Asian	1	1
	White/Caucasian	24	18
	Mixed-race (*e.g.* brown)	107	80
	Black	2	1
	**Total**	134	100
**Age**	**71 years old (mean)**	145	100

Abbreviation: NA, not available.

 The most prevalent comorbidity was hypertension (54%), followed by diabetes (31%). Among neuropsychiatric disorders, depression was the most common (34%). Rapid eye movement (REM) sleep disorder was reported in 42% of patients. All comorbidities are summarized in Table 2. 

**Table 2:** Comorbidities and life habits.

Comorbidities		Patients	Ratio (%)
High Blood Pressure (NA=12)	No	61	46
	Yes	72	54
	**Total**	133	100
Diabetes Mellitus (NA=12)	No	92	69
	Yes	41	31
	**Total**	133	100
Dyslipidemia (NA=13)	No	94	71
	Yes	38	29
	**Total**	132	100
Thyroid dysfunction (NA=12)	No	119	89
	Yes	14	11
	**Total**	133	100
**Life habits**			
Smoking (NA=12)	No	104	81
	Yes	25	19
	**Total**	129	100
Ethylism (NA=14)	No	102	80
	Yes	25	20
	**Total**	127	100
**Psychiatric comorbidities**			
Depression (NA=11)	No	85	65
	Yes	45	34
	**Total**	130	100
Anxiety (NA=11)	No	110	85
	Yes	20	15
	**Total**	130	100
Sleep disorder (NA=44)	No	56	58
	Yes	41	42
	**Total**	97	100

Abbreviation: NA, not available.

 The Mini-Mental State Examination (MMSE)^17 ,18^, Brief Cognitive Screening Battery^19^, and the Pfeffer Questionnaire^20^ were the most frequently used instruments for cognitive assessment. The average MMSE was 18/30 at the first evaluation. The Brief Cognitive Screening Battery showed an average learning score of 5/10, with a delayed recall score of 4/10. The Pfeffer Questionnaire^20^ primarily measured functional activities, with a mean value of 17/30. 

 Additional instruments included: Brief Battery Cognitive Screening (BBCS) Learning — 67 patients/ Delayed recall — 62 patients/ MMSE — baseline: 125 patients; final: 92 patients/ Pfeffer’s Questionnaire of Function Activities (QAF) — 67 patients. 

 Less frequently used instruments were the Neuropsychiatric Inventory^21^, Geriatric Depression Scale^22^, and the Clinical Dementia Rating (CDR)^23^. 

 Most patients were assessed in either the moderate (30%) or severe (33%) stage of dementia. AD was the most frequent diagnosis (31%), followed by frontotemporal dementia (FTD) (14%), vascular cognitive impairment (9%), and Lewy Body dementia (LBD) (6%). Approximately 36% of the sample was classified as having mixed-pathology dementia, in which more than one etiology was suspected. Additionally, 17% of patients had dementia related to other causes, such as alcohol-related dementia, Parkinson’s disease dementia, toxoplasmosis, post-concussion syndrome, schizophrenia, and systemic lupus erythematosus (Table 3). 

**Table 3:** Diagnosis, dementia level, mixed pathology and follow-up length (months).

		Patients	Ratio (%)
**Diagnosis** (NA=19)	Alzheimer disease	37	30
	Others	21	17
	FTD (includes CBS, PSP)	17	14
	MCI	12	10
	Vascular dementia	11	9
	Lewy body disease	8	7
	Incomplete	7	6
	Mood disorder (depression, bipolar disorder)	4	3
	**Total**	122	100
**Dementia level** (CDR) (NA=44)	Mild cognitive impairment	16	16
	Mild dementia	20	21
	Moderate dementia	29	30
	Severe dementia	32	33
	**Total**	97	100
**Suspected mixed pathology** (NA=32)	No	70	64
	Yes	39	36
	**Total**	109	100
**Median follow up time - months** (NA=32)	Female	22 months	
	Male	23 months	

Abbreviation: NA, not available; FTD, frontotemporal dementia; CBS, corticobasal syndrome; PSP, progressive supranuclear palsy; MCI, mild cognitive impairment; CDR, clinical dementia rating. Notes: ^a^Diagnosis: Alzheimer disease and vascular dementia (2%); Subjective cognitive decline (1%); Anxiety(1%).

 Neuroimaging assessments varied in the sample. Sixty percent of patients underwent cranial magnetic resonance imaging (MRI), and 23% underwent computed tomography (CT). Functional neuroimaging exams, such as positron emission tomography (PET) or single-photon emission computed tomography (SPECT), were requested in atypical cases (*e.g.*, early-onset dementias or AD *vs.* FTD). In another study, PET data showed its contribution to dementia diagnosis when the clinical evaluation was inconclusive. PET-CT aided in 21 of 29 cases^24^. Neuroimaging results are summarized in Table 4. 

**Table 4:** Neuroimaging assessment.

		Patients	Ratio (%)
**MRI^a^** (NA=58)	Others	31	36
	Microangiopathy	16	18
	Hippocampal atrophy	14	16
	Normal	10	11
	Biparietal atrophy	6	7
	Frontal atrophy	6	7
	Microbleeds	2	2
	Total	87	100
**CT scan^b^** (NA=111)	Normal	10	29
	Hippocampal atrophy	1	3
	Microangiopathy	8	24
	Biparietal atrophy	1	3
	Frontal atrophy	2	6
	Total	34	100
**^18^FDG PET** (NA=83)	Normal / Inconclusive	24	40
	Suggestive of AD^c^	19	30
	Not suggestive of AD	19	30
	Total	62	100

Abbreviation: NA, not available; FTD, frontotemporal dementia; CBS, corticobasal syndrome; PSP, progressive supranuclear palsy; MCI, mild cognitive impairment; CDR, clinical dementia rating. Notes: ^a^Diagnosis: Alzheimer disease and vascular dementia (2%); Subjective cognitive decline (1%); Anxiety(1%).

Notes: ^a^MR: Hippocampal atrophy and Frontal atrophy (1%); Microangiopathy e Biparietal atrophy (1%); ^b^CT SCAN: Others (32%); Hippocampal atrophy and Frontal atrophy (3%); ^c^hypometabolism predominantly in temporoparietal regions, pre-cuneus and posterior cingulate cortex.

## DISCUSSION

 In the present study, the clinical and epidemiological characteristics of 145 patients treated at a specialized dementia outpatient clinic at a reference center in Recife, Brazil, were highlighted. These numbers provide valuable insight into an underrepresented population in dementia studies. 

 Significant variations were observed in data entry quality, the tests performed, and how medical records were documented. While some information, such as years of education and MMSE scores at the first consultation, was consistently reported, other scales, including the clinical dementia rating (CDR), neuropsychiatric inventory (NPI), or geriatric depression scale (GDS), were less frequently recorded. These inconsistencies limited the study due to the need for uniformity in recorded data. Additionally, the low education level in a substantial portion of the sample, coupled with the high prevalence of moderate and advanced dementia, likely explains the low MMSE scores at initial consultations, consistent with existing literature^4^. Compared to other studies^2^, AD was less representative (31%) in this sample. Since all cases were classified according to current clinical criteria, some patients may have a different underlying pathology from the expected clinical phenotype. Referral bias may explain this, as specialized memory clinics tend to receive more non-AD cases. Moreover, most patients were evaluated in moderate or advanced stages of dementia, which may be explained by delayed access to memory clinics in the overloaded Brazilian public health system and limited availability of specialized centers^25^. 

 In this sample, 51% of participants were divorced, and 34% were widowed. Divorce and widowhood are linked to increased loneliness in old age^26^. Social isolation, a known risk factor for dementia, exacerbates this issue^1^. A study of approximately 7,500 participants from the UK Biobank found that feeling lonely was associated with an approximately 60% higher risk of developing all-cause dementia over nearly 16 years of follow-up. Specifically, loneliness was linked to about a 40% increased risk of AD, an 80% increased risk of vascular dementia, and a 60% increased risk of frontotemporal dementia. Although socioeconomic factors, depression, social isolation, vascular and behavioral risk factors, and genetic predisposition may act as confounders, the association between loneliness and dementia has gained increased interest^27^. 

 Socioeconomic differences and the limited access to education may explain the higher burden of dementia in LMICs. In this study, 15% of patients were illiterate, and 22% had low education (1 to 7 years of schooling). In Recife in 2010, 46.5% of the population was illiterate or had low education^11^. Quality education in childhood and higher levels of education throughout life are known to reduce the risk of dementia^1^. 

 The Brazilian population exhibits a multiethnic background, with contributions from African, indigenous, and white Caucasian descendants^28^. Most participants in this study self-identified as brown. Afro-descendants are at higher risk for dementia in Brazil^4^. Studies indicate that being Black and residing in the North or Northeast regions is associated with poorer access to healthcare^29^. In the UK Biobank, an analysis of approximately 4,700 patients revealed that, even after adjusting for apolipoprotein E (APOE) and AD genetic risk scores, the increased risk for black individuals remains significant. Lifestyle factors may partially explain this heightened risk but do not account for it entirely. Notably, AD risk polymorphisms have a significantly lower frequency in the Black population^30^. 

 Another significant factor is the high prevalence and increasing incidence of public health issues such as hypertension, diabetes, and obesity, particularly in Latin America. Hypertension, a crucial midlife risk factor, was highly prevalent in this sample and is linked to brain volume reduction and increased white matter hyperintensities observed on MRI^31^. Substantial evidence indicates that managing high blood pressure lowers the risk of developing dementia^14^. Diabetes was the second most prevalent comorbidity in this sample, a condition associated with both increased disease duration and severity^1^. 

 The study found that women’s primary caregivers are typically their sons or daughters, whereas men’s primary caregivers are usually their wives. This pattern reflects cultural norms in Latin families, where caregiving roles are often assumed without deep consideration, leading to cultural bias. "Familism" describes the strong sense of loyalty, reciprocity, and solidarity among family members, while "marianism" pertains to the expectation that women will sacrifice themselves for the family’s benefit^32^. 

 Depression was prevalent in this sample (34%). Depression in older adults is associated with cognitive impairment1. The relationship between depression and dementia may be bidirectional; depression can be a risk factor, a prodrome, or a consequence of dementia. Evidence suggests that individuals with depression experience an imbalance in amyloid-beta plaque production and clearance. Additionally, depression reduces neurotrophic factors, which are crucial for synapse development and neuroplasticity^33^. 

 This study has limitations due to its retrospective and descriptive nature. Cognitive and functional assessments were heterogeneous in the medical records, reinforcing the need for harmonized protocols in this and other centers. Additionally, knowing the frequency of patients using cholinesterase inhibitors and/or memantine could provide more information for future reports. It is also recognized that these results may not reflect the reality of other centers in the region, as most patients in this sample had access to specialized evaluation in a tertiary center with proper medical and imaging resources. Characterizing the cognitive profile and number of dementia cases in this context is challenging due to:

- heterogeneous cognitive screening in primary care;
- limited access to resources such as advanced neuroimaging, APOE status, genetic testing, and neuropsychological evaluation;
- referral centers predominantly based in universities;
- a strong stigma surrounding dementia^25^ .

Finally, the strengths of the present study are highlighted. These findings contribute to the limited literature describing dementia centers in underrepresented populations, with data from multiracial Brazilian older adults living in an urban area. The data gathered may set the foundation for collaborations with other centers and prospective studies to improve knowledge of dementia prevalence in Northeast Brazil. 

 In conclusion, in this sample of older Brazilian adults evaluated at our center, most patients had low educational levels, and women were the primary caregivers. MMSE, BBCS, and Pfeffer tests were the most frequently used instruments. Patients were mostly in moderate and advanced stages of dementia, with AD being the most frequent diagnosis, followed by other degenerative conditions such as FTD, vascular dementia, and mixed pathologies. MRI was the most commonly used neuroimaging tool. These findings provide a foundation for longitudinal monitoring with harmonized protocols across centers allowing for better characterization of dementia prevalence in Northeast Brazil. 

## Data Availability

The datasets generated and/or analyzed during the current study are available from the corresponding author upon reasonable request.
